# A Possible Fallacy in Vital Statistics

**Published:** 1917-04

**Authors:** 


**A Monthly Review of Medicine and Surgery**

EDITOR AND PUBLISHER, DR. A. L. BENEDICT, 228 Summer St., corner of Elmwood Ave., Buffalo, (Address for all communications. Please make personal and telephone calls before 1 P. M.)

Third, in age, on the western continent. Independent, but supports professional organizations. News limited mainly to Western N. Y. and Penn.

Subscription $2.00 a year; $3.00 for two years in advance. Changes and errors in address or failure or duplication of delivery, should be reported immediately.

Yearly Volume 72 APRIL, 1917 Number 9

### A Possible Fallacy in Vital Statistics.

We have called attention in the past to the fact that, if the population of any community is fixed at a decennial census and no allowance made for subsequent changes (which are usually for this country in the nature of a fairly regular increase), ratios of deaths, births, etc., for intervening years will tend to show a gradual fictitious increase, jumping back to an approximately correct figure at the next census year. For some years, it has been the custom to correct this obvious error by estimating the rate of increase of the population according to that actually established by the censuses of the previous decade. It occurs to us, that under present conditions, a very considerable error will follow even this attempt at correction. For instance:

From 1900 to 1910, the population of the country increased from 75,994,000 to 91,772,000—an increase of 15,978,000. The immigration for the corresponding period was 8,805,000 though further allowance must be made for the fact that approximately 20-25% of the immigration is balanced by emigration, not to mention ordinary travel. The latter includes a number of non-emigrant aliens, that is persons of foreign birth, leaving the country but not expecting to return permanently to their former homes. This number is nearly equal in many years, to the expected alien emigration and it is, of course, impossible, to say just what per cent. of the non-emigrants return to this country. At any rate, more than half of our increase in population in recent years has been accounted for by the gross immigration.

For the 14 years, 1901-1914, the total immigration was not quite 13,000,000, the average for the last ten years being just about a million. It is interesting to note how the war has affected the problem. In 1914 (through June 30), the total immigration was 1,218,480, for the next two years 326,700 and 298,826, respectively. The number debarred and deported showed absolute decrease but relative increase. The emigrant aliens for the fiscal year ending June 30, 1914, numbered 303,388, for the next two years, 204,074 and 129,765 respectively. The non-emigrant aliens for the three years numbered, respectively, 330,467, 180,100, and 111,942. It is certain that practically all of the non-emigrant aliens of the last two years and a large share of those of the first of these three years have remained abroad. There is no doubt but that the net immigration for the last two years has been practically nothing. Incidentally, it may be said that so far as immigration and emigration as ordinarily understood, are concerned, the balance has been strongly outward and that the ultimate balance has been due mainly to immigration from Canada. So far as we can learn, all the estimates of increase of population made since the middle of 1915, the population for which time was partly based on state censuses and largely on estimates for 5 years' increase since the national census of 1910, are on the basis of a total national increase of about 1,600,000 per year. This increase presupposes a gross immigration of about a million a year and a net immigration, of about 800,000 and also that aliens leaving the country with the expressed intention of returning, actually do return for the most part. So far as we can estimate, we are short about 2 1/3 millions of the suppositious increase from without and we predict that the census of 1920 will show a shortage of about half the increment estimated for the decade from 1910.

